# A Treatise on the Inhalation of the Vapour of Ether for the Prevention of Pain in Surgical Operations

**Published:** 1847-04-01

**Authors:** 


I. A TREATISE ON THE INHALATION OF THE VAPOUR OF ETHER FOR THE PREVENTION OF PAIN IN SURGICAL OPERATIONS, &c. By *James Robinson,* Surgeon Dentist to the Metropolitan Free Hospital. 8vo. pp. 64. Webster, 1847.

II. GAZETTE MEDICALE, 1847.

FEW persons will forget the sensation excited in professional circles, towards the end of last December, by the rapid propagation of the report that

Mr. Liston had succeeded in practising one of the capital operations of surgery, the patient having meanwhile been rendered totally insensible to pain by the inhalation of ether. The incredulity attending the first reception of the news, was, upon its verification, succeeded by an enthusiasm which has ever since held the public and the profession well-nigh spell-bound. Excusable as this is in a matter of such momentous interest, we fear it has led to a too indiscriminate adoption and a too exaggerated estimate of the value of the remedy, and has certainly given rise to practices which are highly derogatory to professional dignity. The theatres of our hospitals have been made the scenes of operative display before crowds of "fashionables, lords, princes, distinguished foreigners," and the like, and the proceedings within their walls chronicled in the columns of the daily press with all the tact, exaggeration, and conventional phraseology of the penny-a-liners; who, we sincerely hope will not be allowed to convert the *entrée*, so injudiciously granted them upon the present occasion, into a precedent. Mr. Robinson, in his pamphlet, tells us that some of the higher classes of society have graciously condescended to try the effects of the ether upon their own persons; and, referring to some experiments he had the honour to make upon Lady Blessington, Prince Bonaparte, the Marquis of Douro, Count d'Orsay, &c. &c., adds,

"I have mentioned these trials to shew that the experiments have not been confined to the poor, but that persons holding a high position in the aristocracy of intellect and rank, have submitted to the inhalation, merely to test its effect: and this is as it should be; as the opinion of such distinguished individuals will naturally favour the practice and lessen the prejudices against it in all classes of society." P. 23.

The "opinion of such distinguished individuals" is literally not worth a straw, and, indeed, *pro tanto* is adverse to the probability of any scientific fact it is given in favour of. It is of this class of persons the ignorant portion of the public and the medical profession have just right to complain. The patrons of every system of quackery and delusion that offers itself, nothing is too preposterous for its credulity, or too absurd for its practical adoption: and, in the place of holding out an example to the less educated members of the community, it falls the victim to duperies, which their poverty or their natural common sense preserve these latter from. In respect to the proceedings of the medical body itself, we fear that some of the remarks, made some time since by the Editor of the *Gazette Medicale*, are still applicable upon this side of the Channel.

"What should be our object; first of all to verify the fact, to establish it in its purely experimental reality, and to lay down with precision its laws and conditions; secondly, to determine its theory and physiological signification. Instead of this, what has been done. We see persons more eager to mingle up their names with whatever this discovery may possess of novelty and popularity than to do anything to render it more useful to science and humanity. Most of the essays hitherto made are imprinted with this unreflecting precipitation. Patients, in whose cases operations might perhaps have been dispensed with, have been sacrificed to this desire to have one's doings talked off; and, instead of the production of precise, well analysed, clearly established results, there is furnished but a vain gratification of public curiosity, and pretexts for the distrust and slanderings of those who are jealous of all progress. Doubtless we must make allowance for that precipitation and enthusiasm which are inseparable from all that is new; but it is to be regretted that thinking men (hommes serieux) should favour this mischievous tendency, in the sole hope of finding their name quoted in a newspaper or hawked about a drawing-room." Jan. 23.

Although objecting to this precipitation, inasmuch as the value of an excellent remedy has so frequently been eventually underrated through the indiscriminate encomia of its original introducer, and believing that some of the distrust such a course is likely to lead to has already began to be felt; we hasten to state that, we yet feel certain that the Ether Inhalation is destined always to occupy an important place in operative surgery, and eventually to be instrumental in combating various diseased conditions of the economy; but whether these great ends are to be accomplished sooner or later, will much depend upon the care with which cases are selected, and the exactitude with which their particulars are detailed, both prior to and subsequent to the employment of the ether, and that particularly when this has seemed to be injurious in its operation. Of details of this kind we are as yet much in want, the mere fact of the abnegation of pain in the immense majority of cases being nearly all that has hitherto been satisfactorily established.

The vastness of this boon can only be appreciated by those familiar with the performance and witnessing of surgical operations. The surgeon, aware of the absolute necessity of compressing the feelings, in order that he might place his intellect at the service of the patient, did in fact a violence to those feelings the amount of which he himself was hardly aware of, and the firmness he thus acquired was mistaken too often by the ignorant for callous indifference acquired by custom. The best answer to this is the eagerness with which the best operating surgeons have embraced this means of mitigating suffering, and the heart-felt delight they manifest at its success. They know that not only is the patient saved indescribable torture during the operation itself, but also the scarcely less acute mental suffering anticipatory of this. They see in this exemption of pain a motive for not too long delaying necessary operations, and a greater chance of recovering from their effects.

However necessary we and others may think it that a greater degree of caution be adopted in the pursuit of this inquiry, we suppose M. *Magendie* is the only person who objects to it *in toto*, and this he does on grounds so peculiarly his own, that we cannot forbear quoting a portion of the observations he addressed to the Academy of Sciences.

"I cannot consent to associate myself in this matter with the kind of enthusiasm I am witness to. What I see most certain in all this is, that surgeons are experimenting upon the human race without knowing what they are doing, or what results they are likely to obtain. Such conduct is not distinguished by all the morality that is desirable. (Loud expressions of dissent). You plunge your patients into a state of drunkenness, for it is nothing but drunkenness (and whether the substance be inspired or taken as a drink little matters), knowing at present nothing exactly concerning intoxication produced by ether, which as yet has not been studied with attention. * * * * * In acting thus upon your patient you deprive him of all consciousness, and place him entirely at the mercy of those around him. Is it a moral action to plunge a woman into a state of drunkenness and render her insensible to all around her? Have you reflected on all that may result from this? In my eyes this new method is liable to such grave inconveniences, that I cannot too loudly protest against the generalization of its employment. Is whether a patient suffers more or less pain a subject fitting for the Academy to employ itself with? When I see our table covered as it is to day with a multitude of apparatus, each more absurd than the other, I must avow I experience a very painful sensation. If you wish to continue your experiments upon the ether, do it logically and give it in the fluid form. The vapour is often attended with serious inconveniences, as witness the precautions we take when allowing patients to inspire other substances, such as prussic acid, chlorine, &c. I know well that, what my brethren are doing is with a philanthropic object, but their experiments should be discountenanced, as they may lead to serious accidents, and I insist upon it are of an immoral tendency."—*Sitting of* 1*st Feb.*

The speaker further pointed out the advantage which the retention of sensibility afforded during the progress of operations involving implication of nervous trunks; and commented upon the unreflecting precipitancy with which the results obtained are published. We can only find space for a small portion of M. *Velpeau's* reply.

"Assuredly the effects produced by ether will not pass without being criticised; but I did not expect such a criticism from M. Magendie, and still less could I have anticipated a protest against experiments from him. The expressions he employs are somewhat ungracious, insinuating as they do that we have undertaken experiments without any precaution, and without knowing what we were in search of. On the contrary, the most exact precautions were taken, and it was not until the possibility of producing insensibility by the ether had already been ascertained that we commenced our trials. Moreover, looking at the thing in a proper light, these are not what ought to be termed experiments.

"M. Magendie declares that it is no great thing to suffer pain, and that a discovery which has only its prevention as its object possesses little interest. But does he think there is nothing besides this pain? Is he aware of the anguish it causes the friends of the patient? He seems to participate in the vulgar opinion that surgeons are hard-hearted and insensible. He is not aware then—yes, he knows it well—of the amount of emotion surgeons suppress when they perform a grave operation! If they do not allow this to become apparent, it is because coolness and apparent impassability are among their most essential possessions. How can we be astonished then that they receive with delight a discovery which produces insensibility without danger?"

Although observations so splenetic and ridiculous as those of M. Magendie are calculated in no-wise to impede the adoption of etherization, numerous facts which have now been collected tend to show that it is by no means so harmless a remedy as at first supposed, and that the indiscriminate extension of its employment is objectionable. That the mishaps which have arisen from its use, are, when we consider the immense number of persons of all temperaments, and under every variety of circumstances to whom the ether has been administered, infinitely less than might *a priori* have been expected from the use of a substance capable of so profoundly modifying the normal conditions of the economy, may be cheerfully allowed: but that death itself in several instances, and a very alarming approach to it in many others, have been the direct consequences of such employment, is now familiarly known—not to speak of various hysterical and convulsive symptoms which have been generated by it, and which, although of minor importance, must be taken into account, when we wish to determine the true therapeutical powers of any medicinal agent. It may be said that ether, in thus in some subjects inducing the worst effects, only acts like various other remedies, which, powerful for good, are yet, injudiciously employed, as productive of evil. But, in regard to most of these substances, we are enabled, by consulting the conditions of the economy and duly regulating the dose, to avoid mischief of this kind: yet this is far from being the case with ether, and even in the recent fatal case at Grantham, the proper precaution of making a preliminary trial of its effects afforded no indication of what was to follow. It is quite true that a more extended and more carefully devised and recorded experience may eventually put us in the possession of the means of guarding against these ill-consequences: but, in the mean time, we contend that this measure should *be far more sparingly employed than at present for the relief of the minor class of pains and ailments.* Above all, means should be devised for rigidly confining this great experiment to professional hands accustomed to the necessary precautions, and for preventing quacks and the public from tampering with so powerful an engine, and one which so used might, from the peculiar effect it produces upon some women, indeed become, what M. Magendie now slanderously says it is, immoral. Already in London, chemist's shops are placarded "Painless Extraction of Teeth," and those of Paris "Ici on Etherize;" and, although the correctional police of the latter capital is armed with sufficient power to suppress any abuse of this kind, we fear that we have in this country no anticipatory means at command, and must wait until a few deaths have been produced, and the cumbrous and uncertain machinery of the coroner's inquest put into force, before any check can be placed upon persons, however ignorant or designing they may be, meddling with this powerful agent.

But limiting the investigation to grave cases, and remembering that the two great effects of etherization are the temporary extinction of pain and great relaxation of the muscular system, surely the field in which its powers may be fairly brought to the test is wide enough, as is sufficiently seen from the mere enumeration of certain affections in which it either has or may be tried, viz. obstinate neuralgia, tic-douloureux, spasmodic diseases, tetanus, hydrophobia, strangulated hernia, dislocations, irritable conditions of the urethra or bladder preventive of sounding, &c. Among the extensions hitherto attempted, that in reference to *obstetrics* is certainly one of the most interesting, as calculated, if applicable to natural labour, to ensure a greater aggregate amount of exemption of suffering than any other. By many, the pains of labour are spoken slightingly of, and certainly in numerous cases neither their duration or their severity call for interference: but in other cases, just as numerous, the suffering from them is truly dreadful, demands for the exercise of our ingenuity for its relief quite as urgently as do most surgical operations, especially as its prolongation is so much more considerable than that produced by these. We hold, however, that the conditions for the safe application of ether are not yet sufficiently ascertained to justify our resorting to it in natural labour, which is usually a process unattended with danger to life, or when such danger is produced this is for the most part induced by the occurrence of hæmorrhage, which, there is some reason to fear, etherization might even favour the continuance of. In the mean time, the results of the trials which Professors Simpson and Dubois have made of this substance in natural, difficult, and instrumental labour, are in the highest degree satisfactory, proving as they do, that, notwithstanding the utter annihilation of pain, the contractions of the uterus and of the abdominal parietes continue active and complete, so that the labours were just as, if not more, promptly terminated; and in one of his cases, in which the child was very suddenly expelled, M. Dubois noted the important fact, that a very unusual degree of relaxation of the perineum took place. The anticipation of utility from etherization in turning, in cases in which from delay the uterus contracts powerfully around the child and impedes the passage of the hand, seems scarcely well-founded; inasmuch as, during the insensibility induced by its operation, the action of that organ continues as vigorous as ever. In considering the propriety of adopting etherization in the practice of midwifery, we must take into account its effects upon the fœtus. As far as the trials of MM. Dubois and Simpson extend, these seem to have been in no wise injurious, although the former observed the fœtal cardiac pulsation temporarily mounted up from 125 to 160.

The discrepancy of opinions as to the *modus operandi* of this new remedy sufficiently proves that we have only approached the first stage of the investigation. Indeed, the phenomena induced are so various and uncertain that their classification is at present impossible. Much of this seemed to be due to the imperfection of the aparatus, the unskilfulness of its employment, or the impurity of the ether: but all these circumstances having been duly provided for, and administered by the same hands and by the same mode, excepting the production of the great end of the abolition of sensibility, the various physiological and pscyhological phenomena have manifested no kind of regularity in their development. One person shall become impassable as the subject on the dissecting-room table, another talk incoherently or mirthfully reply to questions or obey directions, others utter exclamations of pain, of which they afterwards retain no reminiscence of having felt, and others, again, declare they have suffered pain, but felt themselves powerless for its expression. Finally, in not a few, ungovernable violence or convulsive action, quite adverse to the performance of any delicate operation, takes place. With some, an utter oblivion is induced, while others, while undergoing all the apparent tortures of a prolonged dissection, are revelling in the realms of memory and the fields of the imagination. The medical profession, as if in contradiction of M. Magendie's calumnies, has been nowise backward in trying experiments upon its own members, competent to render an account of the phenomena observed: and, in Paris, exhibitions of this kind have been even carried to a ludicrous extent by some of the medical students. The sensations induced are almost universally described as pleasurable, and much resembling those resulting from the early stages of alcoholic intoxication; but the greatest variety of the effects upon self-consciousness, and in the degree in which the recognition of surrounding objects is retained, are reported. With many, the hilarity induced quite equals that produced by the inspiration of the nitrous oxide. M. Jobert and other observers have attempted to indicate three distinct stages in its effects, according to the prolongation of the etherization: 1, that of incoherence, agitation, or delirium, as the case may be; 2, acceleration of the pulse with loss of sensibility and loss of voluntary power; and 3, exhaustion and coldness of the surface. We wish the matter could be thus methodically laid down; but it is quite certain that any of these conditions may be induced in different individuals by very various doses of ether, while others, again, are susceptible of only the first degree to appearance and yet enjoy an immunity from suffering during operations. Even the quickened condition of the pulse and respiration, and the stationary one of the pupil, are by no means invariable criteria of the effect having been produced, which, again, in other cases, may take place prior to their induction.

We consider the analogy between etherization and asphyxia, so strongly insisted on by many of the French Academicians, as defective in many of the conditions usually held necessary to constitute this latter state. Amussat states that, as the result of his experiments upon animals, the blood flowing from the carotid artery was dark or fluid, according to whether the inhalation was pursued or not. M. Renault, detailing those tried at Alfort, stated that such change in the blood only took place when the animals were absolutely deprived of access of air, when, of course, they were literally asphyxiated. M. Laugier and other surgeons deny that any darkening of the arterial blood takes place during operations; but M. Blandin and others affirm that this is the case; and we find the same discrepant testimony, upon a point apparently so easily determined, likewise in this country. M. Lassaigne, analysing samples of venous blood taken from a dog prior to and subsequent to inhalation, found the following proportions to exist:—

| | | |
|---|---|---|
| Prior to Inhalation | Clot .. | 65·46 |
| | Serum.. | 34·54 |
| | | 100·00 |
| After Inhalation .. | Clot .. | 59·69 |
| | Serum.. | 40·31 |
| | | 100·00 |

The serum of the blood after inhalation retained a reddish colour for several days, and its coagulum acquired a somewhat greater consistence. The proportions of fibrine, globules and albumen (allowing for the excess of serum mentioned above) bore a normal relation to each other. A minute portion of the ether, forming about 0,0008 of its mass is absorbed and dissolved by the venous blood.

Various of the French Academicians have instituted series of experiments upon animals for the purpose of determining the mode and order in which the various portions of the cerebro-spinal system are influenced during inhalation. The following are some of the conclusions arrived at by the veteran vivisector, Baron Flourens.

"The action of ether upon the nervous centres follows a given course. It acts first on the cerebral lobes, disturbing the intellect; it acts, secondly, upon the cerebellum, deranging the equilibrium of the movements of the animal; thirdly, it acts upon the medulla spinalis, in which it extinguishes successively the sensory and motor principle; and lastly, upon the medulla oblongata, where arrived, life becomes extinct.

"It is impossible to observe a single case of etherization without being struck with the resemblance the new phenomenon bears to those of asphyxia, and experiments exhibit a real relation between asphyxia and etherization. But in ordinary *asphyxia* the nervous system loses its power under the influence of black blood, of blood deprived of oxygen: but in *etherization* it does so under the direct action of this singular agent. This is really all the difference; for in both there is the same loss of sensation and voluntary motion, and the same, at least temporary persistence of the respiratory movements—in one word, there is the same survival of the medulla oblongata over the spinalis. Etherization exhibits to us the entire mechanism of asphyxia—I mean the *successive death* of the various nervous centres. It isolates, just as mechanical experiments do, the intellectual powers, the co-ordination of the movements, sensibility, motility, life. The isolation of life, this point, this vital knot of the nervous system, forms the most striking fact of the new experiments. In an etherized animal one point alone survives, and while it does so, all the other parts retain at least a *latent life*, and may resume their active life: this point once dying all dies."

We fear much light is not shed upon the subject by these experiments; for certainly no such exact order in the train of symptoms is observed in the human subject. We are yet, however, we repeat, in the very infancy of the investigation, and careful observation, well weighed deduction, and a not too hasty extension of inhalation for the relief of minor ailments, seem to be the indications for some time to come. That we have become possessed of a valuable instrument, not only as a therapeutical agent, but for the solution of more than one interesting physiological problem, we hold to be certain: and it much behoves us not to discredit it in the eyes of the public or the profession, by a too rash and careless employment. One benefit the discovery has conferred medical men must feel keenly, the overthrow of the only tangible pretension of Mesmerism. It was thought by many, that the profession were remiss in not putting its boasted power of relieving pain to the test. Their joyful acceptance of a means unassociated with chicanery or humbug of any kind, amply proves that any backwardness in this particular did not arise from indifference or a want of duly estimating the importance of the subject, but from their scepticism and their unwillingness to mix themselves up with this wretched quackery.

Mr. Robinson's pamphlet contains a brief history of the progress of the discovery from the period of its importation to the date of the publication. In our notice of his former work we commented upon his undue laudation of the American dental practitioners. We are now rejoiced in the opportunity of acknowledging the great obligation the human race is under to one of these—always supposing his claim, now litigated, is finally established.

